# Role of Daily Milk Volume and Period of Lactation in Nutrient Content of Human Milk: Results from a Prospective Study

**DOI:** 10.3390/nu12020421

**Published:** 2020-02-06

**Authors:** Virginie Rigourd, Isabel Lopera, Florina Cata, Guy Benoit, Benedicte Jacquemet, Alexandre Lapillonne

**Affiliations:** 1 Neonatal Department, Necker Enfants Malades Hospital, 75015 Paris, France; virginie.rigourd@aphp.fr (V.R.); isalopera2k@ufl.edu (I.L.); 2 Milk Bank Ile de France, Necker Enfants Malades Hospital, 75015 Paris, France; benedicte.jacquemet@aphp.fr; 3 Neonatal Unit, Centre Hospitalier de Remiremont, 88200 Remiremont, France; florinasasca@yahoo.com; 4 Pharmacology Trousseau Hospital, 75015 Paris, France; guy.benoit@aphp.fr; 5 EHU 7328 PACT, Imagine Institute, Paris Descartes University, 75015 Paris, France

**Keywords:** macronutrients, sodium, human milk, preterm infants

## Abstract

Most studies assessing the macronutrient content of human milk are published retrospectively using analyzers that fail to determine sodium content and do not take into account the role of volume in milk composition. We aimed to describe macronutrient content and sodium content in human milk over time, observe any associations between them, and determine the factors associated with the evolution of milk composition. A prospective, longitudinal, monocentric study was undertaken. Contents of protein, fat, and lactose of 102 milk samples from 40 mothers were determined using a human milk analyzer and that of sodium with a flame spectrophotometer. Milk volumes along with clinical data were recorded. Protein content in the fourth quartile of volume was significantly lower than that in the first three, suggesting the existence of a volume threshold for protein content at approximately 445 mL. After multivariate analysis, it was found that maternal age, average volume, and lactation period remained significantly associated with protein content, maternal age remained significantly associated with fat content, and only average volume with sodium content. In consideration of previous findings along with our data, we suggest that extra care should be taken with fortification for feeding preterm infants when the mother’s milk volume is greater than 400–450 mL.

## 1. Introduction

Human milk is the gold standard for feeding preterm infants, but it needs to be fortified to promote adequate growth. Donor milk is often used in the absence or insufficiency of the mother’s milk. The composition of human milk varies throughout the lactation period as a result of internal and external factors, such as gestational age, maternal body mass index (BMI), smoking status, and even diet [1,2,3,4,5]. Past studies have demonstrated that the duration of lactation is a primary determinant of some nutrient contents, such as proteins and sodium [1,2,6,7,8,9,10]. Other studies have been published qualifying breast milk volume as another determinant of protein and sodium contents [8,11,12].

Unlike the clear associations for protein and sodium, fat content trends are more difficult to discern. Studies on the effect of the lactation period show contradictory findings, with one study stating no observed statistical difference in response to length of time, and another reporting changes in a diurnal pattern [2,7].

Given the complexity of these fluctuations, difficulties may arise with providing nutritionally adequate milk to preterm infants, and targeted fortification may be necessary. Preterm neonates often receive donor milk from hospital milk banks as a means of providing the benefits of human milk. However, it is difficult to customize and tailor human milk to the unique needs of each infant. As a result, some infants face the risk of being undernourished [13,14,15].

The use of commercial infrared milk analyzers enables researchers to study the variation in human milk macronutrients in a more efficient manner [16,17]. By doing so, it provides greater insights into fortification practices and may reduce the incidence of nutritional deficiencies [18,19]. However, these devices do not assess all nutrients, particularly sodium. In fact, because many studies are published retrospectively using these analyzers, they fail to take into account the role of volume in milk composition. In order to provide a more comprehensive account of nutrient content in human milk, we have undertaken a prospective cohort study which aims to: (i.) describe all macronutrient and sodium content in human milk over time, (ii.) observe any associations between them, and (iii.) determine the factors associated with the evolution of milk composition.

## 2. Materials and Methods

This study was a prospective, longitudinal, monocentric investigation. Inclusion criteria were mothers who provided their expressed milk to the milk bank—either because they were breastfeeding their offspring or donating their milk—and who had agreed to participate in the study. The mothers were informed that only spare samples would be used for the study and, if possible, additional samples could be collected over the course of the study. Exclusion criteria were a lack of spare samples, mothers who declined to participate in the study, or produced samples after 6 months of lactation.

### 2.1. Samples of Women’s Milk

The donors’ or mothers’ own breast milk was collected at a regional milk bank. All breast milk samples were collected from birth until 6 months postpartum. They were stored in a refrigerator (+4 °C) for a maximum of 48 h before being frozen at −18 °C. For each sample collected, the mothers were asked to record the volume expressed per 24 h on a milk management sheet.

The milk was pasteurized on a weekly basis. For pasteurization, bottles of frozen milk of a single mother from up to 7 consecutive days were mixed to form a batch that was subsequently pasteurized according to the Holder protocol (62.5 °C for 30 min) before being cooled to 4 °C and then refrozen at −18 °C. On the day of pasteurization, two 5 mL samples from each batch were extracted at pre-pasteurization stages. One sample was sent directly for bacteriological analysis and the duplicate was stored at −18 °C as a control if necessary. The controlled milk samples, if not used, were assigned to our study.

### 2.2. Macronutrient and Sodium Content in Human Milk Samples

The contents of protein, fat, and lactose in the milk were determined using the MIRIS HMA™ analyzer (MIRIS AB, Uppsala, Sweden). This analyzer uses medium-infrared spectroscopy to detect the levels of protein, fat, and lactose based on their respective wavelengths as a function of the intensity of the outgoing radiation [16]. The device provided a calculation of energy using the conversion factors of 9.25, 4.4, and 4 kcal per 100 mL for fat, proteins, and lactose, respectively. The accuracy of this device has been validated by several human milk analytical studies [16] and was performed by the manufacturer at the manufacturer’s premises before release. The analyzer’s stability and repeatability were controlled prior to the study. For this assay, 2 mL of the 5 mL thawed sample was heated to 40 °C and homogenized by manual stirring and sonication. The analyzer was set to measure frozen milk. The apparatus was cleaned every 10 samples, as directed by the manufacturer. Due to inaccuracies in lactose measurements [16], we reported the readings of lactose in the results but did not interpret them. Because of an expected concentration ranging between 5 mmol and 20 mmol per liter, sodium content was assessed by a flame atomic absorption spectrometer (Varian AS 240FS Mulgrave, Australia) equipped with a sample introduction pump system and a deuterium lamp for background correction. The wavelengths and spectral bandwidths (SBWs) used for the analysis were 589.6 nm and 0.2 nm, respectively. Samples were diluted to a hundredth with ultrapure water. Accuracy and reproducibility were verified prior to analysis by a preliminary calibration step with a flame spectrophotometer able to measure sodium content in the 0.0086–0.217 mmol per liter range.

### 2.3. Clinical Data

Maternal data were prospectively collected in an Excel file and included age, gestational age at delivery (GA), type of pregnancy, mode of birth (caesarean or vaginal), smoking status, and prior breastfeeding experience. Since milk from several days was pooled into batches, day of milk collection used for analysis was the average of the first and last days of collection. The daily milk volume produced by each mother was recorded in the designated milk management sheet.

For neonates, birth weight and weight percentile were also recorded in the Excel file. Small for gestational age infants (SGA) were defined by birth weights below the 10th percentile on the female Fenton growth curve (https://peditools.org/fenton2013/index.php).

### 2.4. Ethics

This study was approved by the local ethics committee (i.e., Comité de Protection des Personnes Ile de France II; #13003) and informed consent was obtained from all women who participated in the study.

### 2.5. Statistical Analysis

Statistical analyses were performed by the Minitab^®^ 18 software (Statistical Solutions, Kaysville, UT, USA). Descriptive statistics were calculated for all variables. Mothers were separated into three groups by maternal age: mothers below 30, mothers between 30 and 35, and mothers greater than 35 years of age. When considering multiple pregnancies, twins or triplets were classified as SGA if at least one infant fell below the 10th percentile on the Fenton growth curve and were only counted once. Average volume was divided into quartiles. GA was categorized into three groups: very premature (<32 weeks), moderately premature (32–36 weeks), and at term (>37 weeks). Lactation period was classified into four stages: first week, second week, third week, and greater than three weeks.

Pearson’s correlation coefficient test was used to determine which nutrients were interrelated. Univariate analysis was performed with a chi-square test to identify any possible associations with the recorded variables and proteins, fat, and sodium. A one-way ANOVA was conducted to compare the effect of each factor on the protein, fat, and sodium contents in the milk samples. For any significant differences that existed from ANOVA, post hoc comparisons using the Tukey test were used to identify the conditions in which they occurred. Each factor associated with nutrient content in the univariate analysis with a *p*-value less than 0.1 was included in the multivariate analysis. An ordinal logistic regression test was performed to see which independent variables best predicted each nutrient’s content. *p*-values less than 0.05 were considered significant for all tests.

## 3. Results

### 3.1. Population Characteristics

Forty mothers completed the study, providing 102 milk samples. The descriptive characteristics of the study population are shown in Table 1. Of the 40 mothers who participated in the study, 33 delivered 39 preterm infants (≤28 weeks, *n* = 4; 29–31 weeks, *n* = 13; 32–36 weeks, *n* = 17). There were six mothers who delivered multiple pregnancies, representing 15% of mothers in the sample.

### 3.2. Relationships between Nutrients

The relationships between the contents of the macronutrients and sodium with average milk volume are shown in Table 2. Protein content was significantly positively correlated with both fat (*p* < 0.001) and sodium contents (*p* < 0.001). Milk volume was significantly negatively correlated with both protein and sodium contents (*p* < 0.001), but not with fat.

### 3.3. Factors Associated with Macronutrient and Sodium Content

The associations between clinical factors and macronutrient and sodium contents from the 102 milk samples are reported in Table 3. Maternal age was significantly associated with protein (*p* = 0.001) and fat contents (*p* = 0.009), but not with sodium (*p* = 0.404). Average volume of breastmilk was statistically associated with protein and sodium (*p* < 0.05). Lactation period was only associated with protein content (*p* < 0.001) and multiple pregnancies with sodium content (*p* = 0.035).

**Figure 1:**
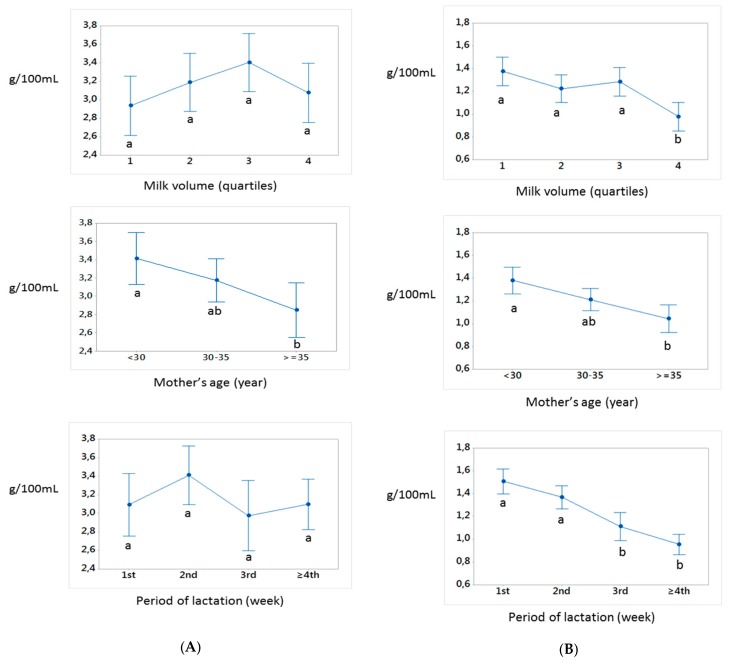

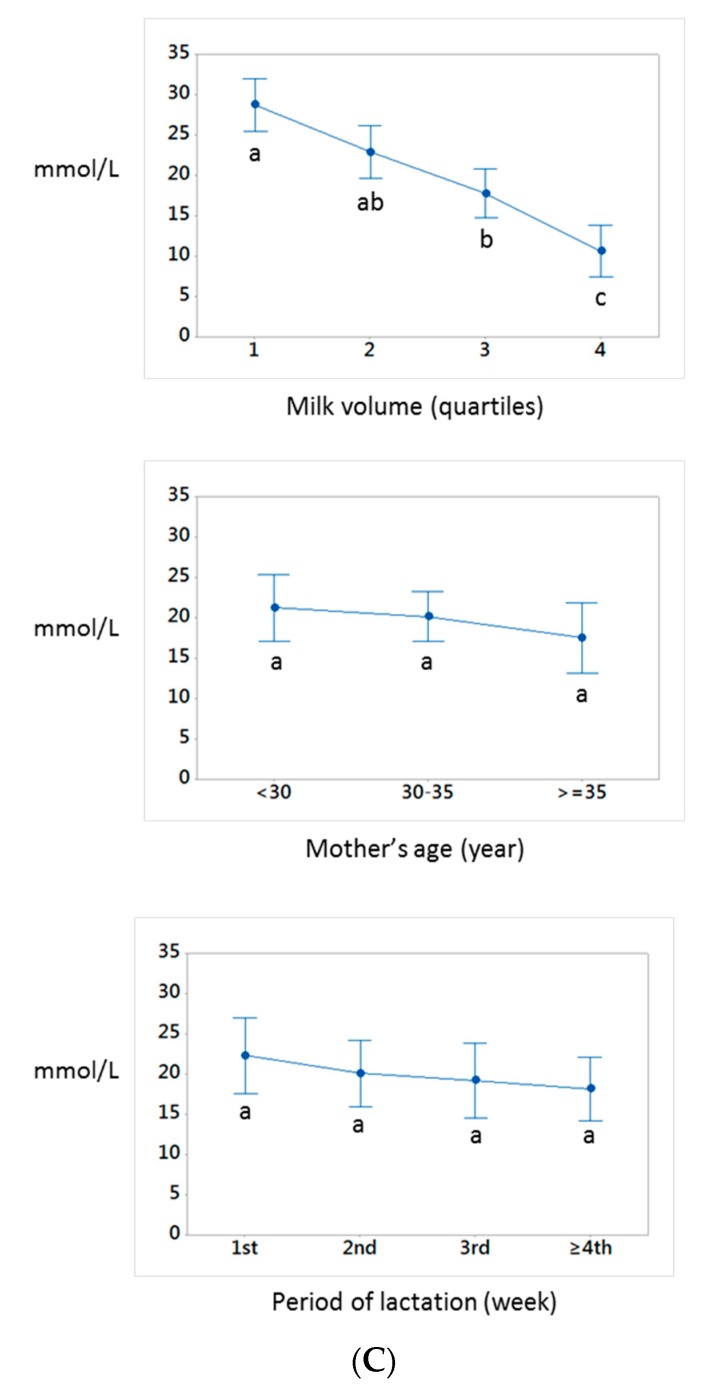
Associations between maternal age, average volume, and lactation period with protein (**A**), fat (**B**), and sodium (**C**) concentrations. Differences in mean ± SD for each nutrient were tested using ANOVA with post hoc Tukey’s test. Different letters (**a**–**c**) confer statistically different mean values.

Mean ± SD maternal age, milk volume, and lactation period are graphically represented with each of the three nutrients in Figure 1 and reported in Supplementary Materials Tables S1–S3. There was a significant association between maternal age and protein and fat contents (*p* < 0.001, *p* = 0.028).

Additionally, volume of breast milk produced per day was significantly associated with protein and sodium (*p* < 0.001), but not with fat (*p* = 0.21). The mean protein content in the fourth quartile of milk volume was significantly lower than that of the first three, suggesting the existence of a volume threshold for protein content at approximately 445 mL. Lactation period was only significantly associated with protein content (*p* < 0.001).

The results from the logistic ordinal regression are summarized in Table 4. Maternal age, average volume, and lactation period remained significantly associated with protein content. Only maternal age remained significantly associated with fat content and only average volume with sodium content.

## 4. Discussion

The present study provides a comprehensive assessment of protein, fat, and sodium contents during a six-month collection period. The samples were categorized in a manner reflective of lactation stages to better observe milk composition changes in response to the tested variables. This is one of the few studies that we are aware of which focused on the relationship between lactation stage, breast milk volume, macronutrient content, and sodium.

The mean protein and fat contents were consistent with previous studies and fell within reported ranges [15,20,21,22]. The mean sodium content was similar or slightly higher than previously reported values, which may be due to the different populations studied, methods used, higher variability of sodium content, or different time periods [4,6].

In this cohort, protein was significantly correlated with both fat and sodium contents. Similar to other studies, our data show that protein and sodium undergo similar negative trends in the first six months of lactation [4,23,24], compounded with them both being significantly associated with average breast milk volume. However, our study provides better insight by demonstrating that both volume and lactation period are independently associated with protein content, while only milk volume remains associated with sodium.

Maternal age also remained the only significant determinant of fat content in our study. Previous studies have documented this association as positive, but only in colostrum, not mature milk, or in populations with a large proportion of overweight and obese women [25,26]. Fat content is indeed known to be the most variable macronutrient in breast milk and has been attributed to either anthropometric or genetic maternal factors, which may explain our differing results from previous studies [27,28,29]. Our study provides new insight into the factors associated with fat content, since our data were adjusted for milk volume, which may be a very important factor in influencing fat concentration in mothers’ milk.

Average breast milk volume remained a significant factor in both protein and sodium contents. Several studies have reported that volumes below a threshold range of 300–400 mL are strongly associated with increases in both nutrients [4,11] in early [8,12] and late stages of lactation [11,30]. Breast milk output below 300 mL/day may be suggestive of weaning, during which protein content rises considerably along with other electrolytes as a result of alterations in tight junction complexes [11]. In our study, we show a possible threshold value for volumes higher than those previously reported at around 440 mL per day. Therefore, in consideration of previous findings along with our data, we suggest that extra care should be taken with the possibility of individualized fortification for feeding preterm infants when the mother’s milk volume is greater than 400–450 mL. Our study also shows that milk volume is significantly lower in mothers who have delivered prematurely. Hypolactation is indeed frequent in mothers who have delivered prematurely, and possibly related to many factors, such as immaturity of the mammary gland, cesarean section, separation after birth, stress related to the prognostic uncertainty, and delay in establishing breast milk.

Unlike other studies that cited gestational age at delivery as a significant predictor of milk composition [7], we found that, after controlling for all significantly associated factors, this relation was not significant. Consistent with recent studies [21,31], we found that the degree of prematurity posed no significant associations with macronutrient and sodium content.

We recognize that our study has several limitations. We were unable to collect information on maternal BMI, as the regional milk bank did not record the height or weight of the mothers. Infant sex was also not accounted for and may have influenced the categorization of SGA infants. Due to possible inaccuracies of lactose measurements with MIRIS HMA™ [16], we were unable to report these results along with energy content as reliable. Another limitation of this study is the lower sample size, particularly for sodium measurements, as not all milk samples were able to be analyzed for this micronutrient, possibly due to having too small a volume for flame spectroscopy analysis.

## 5. Conclusions

Overall, average volume was a strong predictor for protein and sodium levels, and lactation period remained significantly associated with protein content. This study found that maternal age was also a significant predictor of protein and fat contents. In consideration of previous findings along with our data, we suggest that extra care should be taken with fortification of breast milk for feeding preterm infants when daily milk volume is greater than 400–450 mL.

## Figures and Tables

**Table 1:** Clinical characteristics and characteristics of the milk samples.

Maternal Characteristics	*n*	Values
Age (years)	40	32 ± 5.8
Gestational Age at delivery (GA) (weeks)	40	33 ± 3.7
Multiple Pregnancies	40	6 (15%)
Cesarean Section	40	31 (78%)
Smoking Status	40	5 (13%)
Previous Breastfeeding Experience	40	13 (33%)
**Newborn Characteristics**		
Weight (g)	47	1720 ± 834
Weight percentile (%)	47	39 ± 31
**Milk Samples**		
Average Lactation period (days)	102	24 ± 26
Length of Collection (days)	102	6.9 ± 3.4
Average Volume (mL)	102	318 ± 222
Fat (g/100 mL)	102	3.2 ± 0.8
Protein (g/100 mL)	101	1.2 ± 0.4
Lactose (g/100 mL)	102	5.7 ± 0.8
Energy (kcal/L)	102	57 ± 11
Sodium (mmol/L)	80	20 ± 9.7

Data are mean ± standard deviation (SD) or *n* (%).

**Table 2:** Correlations between milk volume, macronutrient, and sodium contents.

	Average Day of Lactation (day)	Average Milk Volume (mL/day)	Proteins (g/100 mL)	Fat (g/100 mL)
Average milk volume (mL/day)	0.393 (<0.001) *			
Proteins (g/100 mL)	−0.555 (<0.001)	−0.451 (<0.001)		
Fat (g/100 mL)	−0.129 (0.195)	0.007 (0.948)	0.395 (<0.001)	
Sodium (mmol/L)	−0.248 (0.027)	−0.672 (0.000)	0.373 (0.001)	−0.137 (0.227)

* Pearson correlation coefficient (*p* value).

**Table 3:** Associations between clinical factors and macronutrient and sodium concentrations expressed in quartiles from 102 milk samples.

Factor	Protein	Lipids	Sodium
Maternal Age	*p* = 0.001	*p* = 0.009	*p* = 0.404
<30 years (*n* = 30)			
30–5 years (*n* = 44)			
≥35 years (*n* = 28)			
GA at delivery	*p* = 0.106	*p* = 0.928	*p* = 0.673
Very premature (*n* = 53)			
Moderate (*n* = 31)			
Term (*n* = 18)			
Multiple Pregnancy (Yes, *n* = 6)	*p* = 0.780	*p* = 0.937	*p* = 0.035
Small for Gestational Age (Yes, *n* = 27)	*p* = 0.816	*p* = 0.390	*p* = 0.880
Cesarean Section (Yes, *n* = 72)	*p* = 0.331	*p* = 0.911	*p* = 0.649
Smoking Status (Yes, *n* = 14)	*p* = 0.162	*p* = 0.191	*p* = 0.146
Previous Breastfeeding Experience (Yes, *n* = 32)	*p* = 0.358	*p* = 0.982	*p* = 0.002
Milk volume	*p* = 0.019	*p* = 0.041	*p* < 0.001
1^st^ quartile (95 ± 32 mL) *			
2^nd^ quartile (191 ± 35 mL) *			
3^rd^ quartile (345 ± 54 mL) *			
4^th^ quartile (646 ± 138 mL) *			
Lactation Period	*p* < 0.001	*p* = 0.921	*p* = 0.518
Week 1 (*n* = 23)			
Week 2 (*n* = 26)			
Week 3 (*n* = 18)			
≥Weeks 4 (*n* = 35)			

* Data are mean ± standard deviation (SD).

**Table 4:** Multivariate analysis by logistic ordinal regression.

Nutrient	Odds Ratio	95% Confidence Interval	*p*
Protein			
Maternal Age	2.59	(1.48; 4.55)	0.001
Milk Volume	1.44	(1.00; 2.08)	0.049
Lactation Period	3.83	(3.50; 5.86)	<0.001
Fat			
Maternal Age	2.67	(1.61; 4.43)	<0.001
Milk Volume	0.73	(0.53; 1.01)	0.06
Sodium			
Multiple Pregnancy	2.43	(0.68; 8.72)	0.172
Previous Breastfeeding Experience	0.68	(0.27; 1.74)	0.425
Milk Volume	4.02	(2.48; 6.51)	<0.001

## Supplementary Materials

The following are available online at https://www.mdpi.com/2072-6643/12/2/421/s1, Table S1: Nutrient contents of human milk according to maternal age, Table S2: Nutrient contents of human milk according to milk volume, Table S3: Nutrient contents of human milk according to period of lactation.
