# Supplementary material for: Role of Daily Milk Volume and Period of Lactation in Nutrient Content of Human Milk: Results from a Prospective Study
Source: Nutrients. 2020 Feb 6;12(2):421. doi: 10.3390/nu12020421 (PMC7071293; doi:10.3390/nu12020421)
Supplement: Supplementary file 1 [file nutrients-12-00421-s001.pdf]

Table S1. Nutrient contents of human milk according to maternal age.

Variable	Maternal age (years)	*n*	Mean	SD	Minimum	Q1	Median	Q3	Maximum
Protein (g/100 mL)	<30	30	1.38	0.28	0.90	1.18	1.40	1.50	2.00
	30-35	43	1.21	0.33	0.40	1.00	1.20	1.40	1.90
	≥35	28	1.04	0.35	0.40	0.80	1.00	1.30	1.80
Fat (g/100 mL)	<30	30	3.41	0.58	2.20	3.10	3.50	3.70	4.70
	30-35	44	3.17	0.98	1.10	2.70	3.10	3.70	6.10
	≥35	28	2.85	0.65	1.40	2.33	2.80	3.10	4.70
Lactose (g/100 mL)	<30	30	5.627	0.246	5.000	5.500	5.600	5.800	6.000
	30-35	44	5.79	1.20	2.40	5.50	5.70	5.80	12.50
	≥35	28	5.55	0.29	4.70	5.40	5.60	5.70	6.10
Energy (kcal/100 mL)	<30	30	60.1	6.3	48.0	56.8	61.0	63.0	73.0
	30-35	44	57.7	14.2	22.0	52.0	56.0	61.8	125.0
	≥35	28	52.9	7.2	38.0	48.0	52.0	57.0	73.0
Sodium (mmol/100 mL)	<30	22	21.3	9.8	10.1	14.4	19.2	24.5	46.0
	30-35	38	20.2	9.4	7.1	12.3	18.5	27.2	42.5
	≥35	20	17.5	10.2	6.7	9.6	14.8	24.0	37.3

Table S2. Nutrient contents of human milk according to milk volume.

Variable	Milk volume (quartiles)	*n*	Mean	SD	Minimum	Q1	Median	Q3	Maximum
Protein (g/100 mL)	1st	25	1.38	0.30	0.70	1.15	1.40	1.65	1.80
	2nd	26	1.22	0.34	0.60	0.90	1.30	1.43	1.90
	3rd	25	1.28	0.31	0.70	1.05	1.30	1.45	2.00
	4th	25	0.98	0.32	0.40	0.80	1.00	1.20	1.60
Fat (g/100 mL)	1st	25	2.94	0.66	1.20	2.45	3.00	3.45	4.20
	2nd	26	3.19	0.83	1.50	2.70	3.00	3.58	5.60
	3rd	26	3.40	0.94	1.40	3.10	3.45	3.80	6.10
	4th	25	3.08	0.79	1.10	2.65	3.00	3.65	4.70
Lactose (g/100 mL)	1st	25	5.54	0.27	5.10	5.30	5.50	5.70	6.00
	2nd	26	5.56	0.33	4.70	5.35	5.60	5.80	6.30
	3rd	26	6.01	1.38	5.40	5.60	5.65	5.90	12.50
	4th	25	5.58	0.69	2.40	5.50	5.70	5.80	6.10

Energy (kcal/100mL)	1st	25	55.4	6.7	40.0	50.0	56.0	61.0	66.0
	2nd	26	56.9	9.1	41.0	51.3	56.0	61.3	84.0
	3rd	26	61.2	15.3	38.0	55.8	60.0	63.3	125.0
	4th	25	54.6	10.0	22.0	50.0	56.0	62.0	73.0
Sodium (mmol/100mL)	1st	19	28.8	8.8	14.4	21.2	31.7	36.0	42.5
	2nd	19	22.9	8.9	11.8	15.9	20.0	26.5	46.0
	3rd	22	17.8	6.3	9.5	12.8	17.4	20.3	32.9
	4th	20	10.6	3.8	6.7	7.1	10.2	12.8	20.8

Table S3. Nutrient contents of human milk according to period of lactation.

Variable	Period of lactation (weeks)	*n*	Mean	SD	Minimum	Q1	Median	Q3	Maximum
Protein (g/100mL)	1st	23	1.51	0.27	0.80	1.40	1.50	1.70	2.00
	2nd	26	1.37	0.23	1.00	1.20	1.30	1.50	1.90
	3rd	18	1.11	0.27	0.40	0.98	1.10	1.25	1.50
	≥4th	34	0.95	0.27	0.40	0.80	0.95	1.10	1.80
Fat (g/100mL)	1st	23	3.09	0.74	1.20	2.70	3.10	3.60	4.70
	2nd	26	3.41	0.81	2.10	2.78	3.35	3.80	5.60
	3rd	18	2.97	0.79	1.10	2.55	3.05	3.55	4.00
	≥4th	35	3.10	0.88	1.40	2.70	3.10	3.50	6.10
Lactose (g/100mL)	1st	23	5.51	0.29	4.70	5.40	5.60	5.60	6.10
	2nd	26	5.74	0.47	5.00	5.40	5.75	5.90	7.50
	3rd	18	5.44	0.79	2.40	5.38	5.70	5.70	6.00
	≥4th	35	5.85	1.17	5.20	5.60	5.70	5.80	12.50
Energy (kcal/100mL)	1st	23	57.6	8.0	40.0	51.0	57.0	62.0	73.0
	2nd	26	60.0	8.5	47.0	53.0	59.5	64.3	84.0
	3rd	18	53.9	10.1	22.0	51.3	56.0	60.3	65.0
	≥4th	35	56.2	14.0	38.0	50.0	54.0	60.0	125.0
Sodium (mmol/100mL)	1st	17	22.3	9.7	9.6	14.0	20.0	27.9	42.5
	2nd	22	20.1	9.4	9.9	13.0	16.9	24.5	46.0
	3rd	17	19.2	9.8	7.1	11.9	15.6	27.5	36.7
	≥4th	24	18.2	10.2	6.7	8.9	17.9	25.1	42.5
